# A pragmatic approach to the diagnosis of inborn errors of metabolism in developing countries

**DOI:** 10.4102/ajlm.v12i1.1946

**Published:** 2023-05-30

**Authors:** John I. Anetor, Bose E. Orimadegun, Gloria O. Anetor

**Affiliations:** 1 Department of Chemical Pathology, Faculty of Basic Medical Sciences, University of Ibadan, Ibadan, Nigeria; 2 Department of Public Health Science, Faculty of Health Sciences, National Open University of Nigeria, Abuja, Nigeria

**Keywords:** basic laboratory investigations, developing countries, inborn errors of metabolism, health education, optimisation of treatment

## Abstract

Inborn errors of metabolism (IEM) are a group of genetically derived diseases that are individually rare but collectively common and can be very severe. While high-income countries usually employ modern scientific technologies like tandem mass spectrometry for IEM investigation, these disorders are, in contrast, only rarely screened for in developing countries due to misconceptions that the required facilities are beyond the reach of these countries. This paper attempts to educate scientists and clinicians in developing countries on low-technology IEM screening methods that only require moderate facilities. Although a definitive diagnosis of IEM may require specialised laboratory investigations and attendant interpretation, in most cases, the basic facilities available in the average clinical chemistry laboratory in developing countries can allow the early detection of IEM. This early detection would facilitate critical early decision making, thus leading to better management, optimised treatment, and reduced morbidity and or mortality of IEM in these resource-limited countries. With this approach, a few referral centres for confirmatory investigation, comparable to those existing in developed countries, could be established. This can be integrated into creative health education for healthcare professionals and families who have individuals with IEM.

**What this study adds:**

IEMs are important enough that every country, developed or developing, should have screening plans and basic laboratory facilities that are adequate for initial IEM diagnosis. No country should therefore give up on testing for IEMs on the excuse of a paucity of advanced facilities.

## Introduction

Inborn errors of metabolism (IEM), also referred to as inherited metabolic disorders, are mutational disorders that impair the function of proteins in every way (mostly enzymatic functions). Archibald Garrod, who was the first to explain the relationship between genotype and phenotype, helped bring attention to IEM through his pioneering work on one of the first types of IEM discovered – alkaptonuria.^1,2^ His work helped to elucidate IEM, thus paving the way for the realisation that every aspect of human anatomy and physiology is determined by biochemical reactions catalysed by specific enzymes, and that enzymes are determined by genetic constitution. Since then, several additional types of IEM have been described, and many more are being investigated (Table 1).^3^ Although IEMs are quite common collectively, individual IEM disorders are rare. The most frequently encountered IEMs are monogenic forms.^4,5^ In most cases, IEMs are autosomal recessive.

**TABLE 1:** Types of inborn errors of metabolism commonly investigated using laboratory procedures.

Inherited metabolic disorder	Examples
Amino acid metabolism	Alkaptonuria, phenylketonuria, maple syrup urine disease
Congenital disorders of carbohydrate metabolism	Von Gierke’s disease (glucose-1-phosphatase deficiency), galactosaemia
Disorders of cholesterol biosynthesis	Familial hypercholesterolaemia
Co-factor and vitamin metabolism	Biopterin
Fatty acid metabolism	Medium and long-chain fatty acid metabolism
Organic acid disorders	Branched chain aminoaciduria
Porphyrin metabolism	Acute intermittent porphyrias
Purine and pyrimidine metabolism	Lesch-Nyhan syndrome
Urea cycle disorders	Citrullinemia, ornithine transcarbamylase disorder
Metal storage disorders	Haemochromatosis, Wilson’s disease

*Source*: Hortin GL. Amino Acids, Peptides and Proteins. In: Burtis CA, Bruns DE, editors. Tietz Fundamentals of Clinical Chemistry and Molecular Diagnostics. St. Louis, Missouri: Saunders, Elsevier Inc.; 2015. pp. 286–317.

According to the 2003 Institute of Medicine report ‘Reducing Birth Defects: Meeting the Challenge in the Developing World’,^6^ birth defects are the leading cause of infant mortality in many regions of developing countries.^7^ In these countries, malnutrition, poverty, disease, and lack of access to healthcare elevate the prenatal risk of birth defects and impose enormous personal and societal consequences. Experts in developed countries often have similar concerns because birth defects are also the highest single cause of infant death in many regions.^8^ There is a link between birth defects and genetic abnormalities such as IEMs,^7^ and environmental and genetic factors are inextricably linked. As these gene-environment interactions are important in IEM, people in developing countries are at a greater risk of developing an IEM.^9^ This highlights the need for scientists and doctors to prioritise IEM detection, management, and prevention through research aimed at identifying environmental red flags. A holistic and integrated approach is also required for the effective investigation and treatment of IEM. To achieve this, collaboration across the distinct disciplines of genetics, evolutionary and developmental biology, environmental science, and sociology is necessary.^7^

This article is, in part, an extended response one of the authors gave at a laboratory medicine congress in North Africa in late 2019, where a questioner inferred that IEMs are not screened for or diagnosed in developing nations because the needed specialist laboratory facilities are not available. Although a recent report from Colombia (a developing country) by Echeverri et al.^10^ outlines the activities in their reference centre that support the questioner’s position, their diagnostic approach is not pragmatic or applicable to most developing countries but may apply to referral centres in developing countries. Nevertheless, they did make a notable point about the importance of health education for both health and non-health professionals, who are critical and underappreciated in the field of IEM. A recent report by Civallero, De Kremer and Giugliani^11^ proposed the use of a few simple and affordable instruments such as spectrophotometer and gas chromatography-mass spectrometry for IEM investigations, as these can give an enormous amount of information that can be adapted to the needs of health facilities in developing countries. This approach contrasts that of Echeverri et al.^10^ and may be more relevant to health facilities in developing countries. The Mayo Clinic has also outlined another diagnostic approach that can be modified for the qualitative and quantitative determination of diagnostic markers of IEM (Table 2).^12^

**TABLE 2:** General methods for the qualitative and quantitative determination of diagnostic markers of inborn errors of metabolism.

Methods	Example of disorder investigated
Thin-layer chromatography	Amino acid disorders
High-performance liquid chromatography	Amino acid disorders
Ion exchange chromatography	Amino acid disorders
Gas chromatography	Organic acid disorders
Tandem mass spectrometry	Many IEMs
Liquid chromatography-mass spectrometry	Organic acid disorders
Colorimetric/fluorometric methods	Many IEMs
Enzyme-linked immunosorbent assay	Organic acid disorders
Gas chromatography-mass spectrometry	Organic acid disorders
Electrophoresis, including acrylamide gel electrophoresis	Amino acid disorders

*Source*: Mayo Clinic Medical Laboratories. Biochemical genetics – Inborn errors of metabolism. Rochester, MN: Mayo Foundation for Medical Education and Research; 2011.

IEMs, inborn errors of metabolism.

Clinically, IEM can be grouped into three categories, namely, disorders that cause intoxication, disorders that affect energy metabolism, and disorders that affect complex molecules.^13,14^ In disorders of intoxication, metabolites accumulate at toxic concentrations and the accumulating metabolites are usually proximal to the metabolic block. Consequently, identifying these metabolites provides a basis for diagnosis using basic laboratory facilities, which are readily affordable in resource-limited settings. Therefore, this article aims to raise awareness of basic investigations that can detect IEM early and do not require expensive or sophisticated equipment commonly used in developed countries.

A quasi-systematic review was conducted where the Science Citation Index and PubMed databases were searched, independent of date of publication, for articles on IEM. We prioritised articles that were published in developing countries and articles published by leading authorities and institutions. We also included the early lectures of the ‘Father of Inborn Errors of Metabolism’, Archibald Garrod.^1,2^ We conducted a limited synthesis of all retrieved articles and found evidence of the paucity of investigations of IEM in developing countries due to the lack of facilities, which we have tried to address in this article. A major limitation of our approach is that it only provides a cursory overview of the topic.

## Signs and symptoms of inherited metabolic disorders

There are some common signs and symptoms that raise the index of suspicion of IEM.^15^ These include acute life-threatening crisis, lethargy or coma with or without a symptom-free period, seizures, persistent vomiting, respiratory distress syndrome (apnoea), poor feeding/failure to thrive, hypotonicity/hypertonicity (ataxia, posturing), hepatosplenomegaly/jaundice, dysmorphic features (facial coarsening), macroglossia, ocular features (cataract, corneal clouding, abnormal eye movements), coarse hair with abnormal texture, abnormal odour of urine/body, loss of developmental milestones, unusual responses to fasting and intercurrent infection (von Gierke’s disease, type 1 glycogenosis), and fluctuating neurological status.

## Basic methods for the laboratory investigation of inborn errors of metabolism

### Alkaptonuria

Alkaptonuria is one of the earliest IEMs described by Garrod.^2^ This is a benign disorder that poses no danger to life except for ochronosis (darkening) of the cartilage, and patients with this condition are prone to arthritis from the fourth decade of life. Alkaptonuria is characterised by the darkening of standing urine, and a simple Benedict’s reaction on the urine sample will give a positive reaction. All that is required for this test is copper sulphate in an alkaline medium, which is inexpensive.

### Phenylketonuria

Some basic investigations can also aid in the early detection of phenylketonuria, which is one of the most well-known and potentially fatal IEMs that is associated with profound intelligence quotient loss and severe mental retardation (MR). Phenylketonuria can easily be diagnosed using the FeCl_3_ test (on which the Phenistix test is based) that requires only a 10% FeCl_3_ solution, the Guthrie test, amino acid analysis using high-performance liquid chromatography or tandem mass spectrometry. The latter two methods may be expensive but could be made available in a referral centre.

The Guthrie screening test for increased phenylalanine in serum is one alternative to specialised high-performance liquid chromatography for amino acid analysis that is within the reach of many laboratories in developing countries. It is a microbiological test that relies on phenylalanine’s ability to counteract the effects of a metabolic antagonist on the growth of a *Bacillus subtilis* strain that requires phenylalanine as a growth factor. The test procedure involves the suspension of *B. subtilis* spores in an agar medium containing a minimum amount of growth nutrients plus a fixed amount of the metabolic antagonist, β-2-thienylalanine, which is a non-protein amino acid similar in structure to phenylalanine. When the infant is 6 days old (or as soon as possible if the baby is being treated with antibiotics that interfere with the test), blood is drawn by heel prick onto filter paper, dried, and sent to the laboratory. These blood spots are cut into uniform discs and then placed on agar plates inoculated with the *B. subtilis* isolate, with the only source of phenylalanine being the blood spot. The blood spot discs are compared to discs containing known amounts of phenylalanine. If the serum contains a high level of phenylalanine, the phenylalanine will diffuse from the sample disc into the bacterial medium to counteract the inhibitory effect of thienylalanine, resulting in a ring of bacterial growth around the disc. If the phenylalanine level is less than 2 mg/dL, the growth inhibition will not be overcome, and no bacterial growth will be observed. As false positives can occur, positive results should be confirmed using a chemical method or chromatography. To avoid false positive results due to liver immaturity, tests should not be performed on premature infants or full-term babies immediately after birth. The infant should also be on a phenylalanine-containing diet for at least 24 h before the sample is collected.^3^

### Maple syrup urine disease

In maple syrup urine disease, there is a deficiency of the branched chain α-ketoacid dehydrogenase enzyme that decarboxylates the branched chain amino acids (leucine, isoleucine, and valine). This results in the accumulation of these amino acids and their metabolites (a-ketoacids) in the blood.

Maple syrup urine disease is a severe IEM that begins in the first year of life and progresses to severe MR, acidosis, coma, and death within 5 years if left untreated. As an alternative to tandem mass spectrometry and high-performance liquid chromatography analysis of organic acids, maple syrup urine disease can easily be detected in the laboratory using the Rothera’s test. This requires a simple sodium nitroprusside reagent (a common reagent in most clinical chemistry laboratories) and ammonium hydroxide. When mixed and pulverised, the test retains its positive reaction, unlike in cases of metabolic derangement such as type 1 diabetes mellitus (diabetes ketoacidosis), where the ketone bodies (acetoacetate, acetone, and 3-hydroxy butyrate) are responsible for the positive reaction. The clinical observation of the characteristic offensive odour of the urine (isovaleric aciduria), which may be the first suggestive sign of abnormal metabolite excretion, may be an indication for this investigation.

### Alpha-1-antitrypsin deficiency

Another IEM that can readily be investigated with the moderate facilities available in most developing countries, is alpha-1-antitrypsin deficiency. Alpha-1-antitrypsin is an antiprotease synthesised in the liver, and its deficiency arises from point mutations in the SERPINA1 gene (single amino acid substitution), with over 70 alleles described.^16^ The normal genotype is piMM, and piMZ is the heterozygous genotype for the Z gene. Alpha-1-antitrypsin deficiency frequently arises from piZZ (the homozygous genotype for the Z gene).^17^ This genetic defect causes the protein to form aggregates in the liver that cannot be excreted, thus causing liver damage. The absence of its antiprotease activity also causes emphysema and cirrhosis in children. Although alpha-1-antitrypsin deficiency is commonly investigated using polymerase chain reaction in advanced laboratories, the combination of traditional liver function tests and cellulose acetate serum protein electrophoresis, when the α-band is either significantly reduced or completely absent, is highly suggestive.

### Wilson’s disease

Wilson’s disease (hepatolenticular degeneration) is another IEM that can easily be investigated in a basic clinical laboratory. The disease is due to a mutation in the ATP7B gene, which encodes the copper-transporting protein, ceruloplasmin. Wilson’s disease is easily identified by Kayser Fleischer’s rings (almond-coloured rings around the cornea). In the laboratory, Wilson’s disease can be diagnosed by determining serum copper levels using simple flame atomic absorption spectrophotometry and ceruloplasmin by standard spectrophotometric methods. The result will show low ceruloplasmin concentrations and serum copper levels accompanied by massive copper excretion in the urine. If necessary, a liver biopsy can be done to detect excessive amounts of copper in the liver.

### Cystic fibrosis

Cystic fibrosis is a common IEM that affects one out of every 2500 live births in the United Kingdom.^18^ Cystic fibrosis is inherited in a recessive Mendelian pattern, and individuals with this disease experience a generalised exocrine secretion disorder that is characterised by abnormally viscous secretions. The functional defect is caused by the cystic fibrosis transmembrane conductance regulator protein, which controls transmembrane chloride transport.^19^ Surprisingly, cystic fibrosis patients who do not have functional copies of the protein have a slew of lung and digestive problems.^20^ Symptoms include recurrent respiratory infections, irreversible lung disease, and pancreatic insufficiency leading to malabsorption. In neonates, intestinal obstruction can occur due to the increased viscosity of faecal material, which is useful in laboratories for disease screening.

Cystic fibrosis can be identified clinically through slit-lamp examination of affected patients’ eyes, which may reveal pathognomonic crystals of cystine in the cornea. This corneal defect can cause photophobia, beginning around the age of two. Cystic fibrosis can be screened for using immunoreactive trypsin in blood. Also, faecal pancreatic elastase-1 is the most widely used test to diagnose pancreatic insufficiency in people with cystic fibrosis. Sweat electrolyte analysis is used to make the final diagnosis; when sweat chloride level is less than 30 mmol/L, cystic fibrosis is not likely, but when it is greater than or equal to 60 mmol/L, it is diagnostic.^21^ Pilocarpine iontophoresis (using an electric device to stimulate sweating) is also a standard test used in the diagnosis of cystic fibrosis.

### Galactosaemia

Galactosaemia is caused by a lack of galactose-1-phosphate uridyltransferase, a rate-limiting enzyme that causes hypoglycaemia. This leads to increased levels of galactose-1-phosphate due to blockage of the typical metabolic pathway, and this may lead to alternative metabolism and direct tissue damage.

Some key biochemical features of galactosaemia include impaired bilirubin uptake and conjugation, increased unconjugated hyperbilirubinemia, hepatomegaly, jaundice, severe MR, accumulation of free galactose, and deposition of galactose-1-phosphate in renal tubules with attendant renal damage and associated generalised aminoaciduria. Simple and practical tests that can be used to investigate galactosaemia include Benedict’s test to detect the presence of reducing substances, thin-layer chromatography to determine the presence of galactose in urine, and galactose-1-phosphate uridyltransferase enzyme assay to detect elevated blood galactose. Amniocentesis may be used for prenatal diagnosis, and the galactose tolerance test may be performed if the expected rise in the blood glucose level after galactose administration is not observed. In advanced laboratories, molecular analysis is available.^22^

The diagnosis of galactosaemia is well within the capabilities of most basic medical facilities and the treatment is simple, only requiring the elimination of galactose from the diet.

### Von Gierke’s disease

In Von Gierke’s disease, there is deficiency of glucose-6-phosphatase, an enzyme that splits glucose from glucose-6-phosphate (Glucose-6-PO_4_ + G-6-Phosphatase → Glucose + PO_4_).

Owing to this metabolic block, glucose-6-phosphate accumulates in the liver. The presence of fasting hypoglycaemia and hepatomegaly are strongly suggestive of von Gierke’s disease and clearly distinguishes it from other glycogenoses such as Pompe’s, McArdle’s, and many others.

### Urea cycle disorders

#### Type 1 hyperammonaemia

Type 1 hyperammonaemia is one of the common urea cycle disorders (UCDs). It is an autosomal recessive disorder that is caused by a deficiency of the carbamoyl phosphate synthetase 1 enzyme. The main biochemical characteristics of UCDs are extremely high blood ammonia levels, MR, and a low urea level. Because of the MR that comes with this metabolic disorder, early detection is critical, and the diagnosis is within the capabilities of a typical laboratory. To screen for type 1 hyperammonaemia, advanced methods such as amino acid analysis using high-performance liquid chromatography or tandem mass spectrometry may be required.

In a resource-limited medical facility, blood gas and electrolyte abnormalities are important for detecting UCDs. The presence of reduced blood urea levels, increased ammonia levels, elevated transaminase (alanine transaminase and aspartate transaminase) activity, and coagulopathy (increased prothrombin time and partial thromboplastin time) should be strongly suggestive of a urea cycle defect and risk of encephalopathy and should be sufficient basis for referring patients for specialist investigation and initiating early patient management.

#### Hyperammonaemia type II

The biochemical lesion of hyperammonaemia type II is a deficiency of the ornithine transcarbamoyl transferase enzyme, and the inheritance pattern is X-linked. The key features of this disorder include elevated ammonia levels in the blood, cerebrospinal fluid and urine, as well as orotic aciduria caused by carbamoyl phosphate channelling into pyrimidine synthesis.

Hyperammonaemia is caused by a lack of any of the urea cycle enzymes. If the metabolic block occurs in one of the earlier steps of the urea cycle, ammonia, which is neurotoxic, accumulates and causes a more severe condition. Conversely, the absence of enzymes catalysing reactions in the later stages of the urea cycle results in the accumulation of less toxic intermediates and less severe symptoms. Hence, the exact point of the metabolic block needs to be identified as it determines the prognosis. Because the brain is sensitive to ammonia, hyperammonaemia, as well as elevated ammonia levels in other body fluids, causes toxic symptoms and neurological damage. Understanding the situation is critical because it guides treatment, which primarily consists of a low-protein diet and frequent small feeds. Hippuric acid formation (conjugation product between glycine and benzoyl is a detoxification step) or phenylacetyl glycine can also eliminate amino nitrogen.

#### Citrullinaemia

Citrullinaemia is another example of UCDs. Citrullinaemia is caused by arginosuccinate synthetase deficiency, which results in elevated ammonia and citrulline levels in the blood. Breast milk should be avoided in this condition because it contains high amounts of citrulline.

#### Hyperornithinaemia

Another urea cycle enzyme deficiency is hyperornithinaemia, which is an ornithine transport defect. It is an autosomal recessive condition in which ammonia and ornithine levels in the blood are elevated.

#### Hyperargininaemia

Hyperargininaemia, which results from arginase deficiency and causes arginine accumulation in the blood and cerebrospinal fluid, is another metabolic abnormality seen in UCDs. It is worth noting that in this condition, cysteine and lysine are excreted in the urine instead of arginine. Mass spectrometry can be used to screen for hyperargininaemia.

## Purine metabolism disorder

Purine metabolism disorder is best illustrated by Lesch-Nyhan’s (L-N) syndrome, an X-linked inherited purine metabolism disorder. The biochemical lesion is caused by a hypoxanthine-guanine phosphoribosyltransferase deficiency, which impairs the purine salvage pathway. This results in the accumulation of phosphoribosyl pyrophosphate, an intermediate in the purine synthetic pathway, and other purine synthetic cycle intermediates in the biosynthetic pathway. Hypoxanthine-guanine phosphoribosyltransferase activity is normal in the brain and at low levels in the liver and spleen.^23^ Some of the neurological manifestations of L-N syndrome are thought to be due to the toxicity of the purine degradation products to the developing central nervous system or the absence of hypoxanthine-guanine phosphoribosyltransferase, which results in an imbalance in adenine and guanine nucleotides at a critical developmental phase for the infant.^24^

Self-mutilation, MR, raised uric acid levels (described as petit gout), nephrolithiasis (renal stone), and gout later in life are the main characteristics of L-N syndrome. The elevated uric acid level in relation to the patient’s age and other clinical manifestations, such as MR, are sufficient indications for L-N syndrome diagnosis in resource-limited countries. Molecular investigation for confirmation may be requested from referral centres.

### Tangier’s disease

Tangier’s disease is one of the common IEMs of lipid metabolism. Tangier’s disease is an autosomal dominant metabolic disorder that derives from the deficiency of adenosine triphosphate-binding cassette transporter-1. The key biochemical and clinical characteristics of Tangier’s disease include defective efflux of cholesterol from cells, reduction in high-density lipoprotein levels in the blood, absence of the α-band in serum protein electrophoresis, accumulation of cholesterol esters in tissues, presence of large orange-yellow tonsils, and muscle atrophy associated with recurrent neuropathies and atherosclerosis. The aforementioned features of the disease are sufficient indicators for the early detection of Tangier’s disease before the onset of the later features, such as neuropathy and atherosclerosis.

### Porphyrias

The porphyrias are a group of IEMs associated with the absence of genes encoding enzymes that catalyse reactions in the haem biosynthetic pathway (porphyria means purple). The porphyrias are characterised by increased production and excretion of porphyrin precursors (delta aminolaevulinic acid and porphobilinogen). Most are inherited as autosomal dominant traits and are classified into three broad groups: hepatic porphyrias, erythropoietic porphyrias, and combined erythropoietic and hepatic abnormalities. The classification is based on the major site where the enzyme deficiency is manifested. The clinical manifestations of porphyrias vary and are not usually associated with anaemia. One of the most common clinical manifestations is acute intermittent porphyria, which is an inherited genetic disorder.^25^

The biochemical lesion in acute intermittent porphyria is a lack of porphobilinogen deaminase (uroporphyrinogen-1-synthetase), which causes a secondary increase in aminolaevulinic acid synthase activity (negative feedback mechanism abolished). Delta aminolaevulinic acid and porphobilinogen, two key intermediates in the haem biosynthetic pathway, are elevated in blood and urine of patients with porphyrias. The porphobilinogen assay should be conducted using fresh urine samples transported in dark bottles. The key reagent required for porphobilinogen is p-dimethylaminobenzaldehyde (Ehrlich’s reaction), which is commonly employed in testing for urobilinogen, a basic test conducted in all laboratories. Elevated urinary porphobilinogen excretion confirms the presence of hepatic porphyria. If porphobilinogen excretion exceeds aminolaevulinic acid excretion, lead poisoning can be ruled out.^26,27^

Acute abdominal pain and neurological manifestations, such as sensory and motor disturbances, including confusion and agitation, are among the clinical symptoms that may be intermittent and vague (dubbed ‘little imitators’). A simple ultraviolet fluorescence test with a Wood’s lamp to demonstrate porphyrin is also useful and easily accessible.

### Congenital hypothyroidism

All newborns in every hospital should be screened for congenital hypothyroidism before they are discharged. Congenital hypothyroidism is a clinically significant disorder, especially due to its adverse neurological outcome. This adverse neurological outcome can however be easily reversed if detected early. Most infants are asymptomatic at birth because thyroxin from the maternal circulation diffuses across the placenta. Congenital hypothyroidism may be suggested by clinical features such as lethargy, umbilical hernia, slow movement, hoarse cry, macroglossia, hypothermia, and hypotonia.^28^ A simple panel of thyroid function tests, especially thyroid-stimulating hormone, are useful investigations to confirm these clinical findings where they manifest. The thyroid-stimulating hormone value is particularly important as it is very sensitive.^29^

### Congenital adrenal hyperplasia

Congenital adrenal hyperplasia is an inherited endocrinopathy caused by the genetic absence of certain enzymes involved in steroidogenesis, particularly the cortisol pathway. Due to the lack of a negative feedback mechanism, this biochemical lesion results in the accumulation of intermediates, some of which are potent androgens such as 17-hydroxyprogesterone, which accounts for virilism and genital ambiguity in affected patients. The most common of these enzyme deficiencies is a 21-hydroxylase deficiency. The enzyme responsible for producing the mineralocorticoid aldosterone may also be altered, which could explain the salt-loss presentation (hyponatraemia and hyperkalaemia). Some of the key clinical and biochemical features include precocious growth, increased testosterone, increased 17-hydroxyprogesterone, suboptimal cortisol level with basal and adrenocorticotropic hormone stimulation tests, small testes, and shock.

Though genetic tests like karyotyping may be used to detect congenital adrenal hyperplasia, basic tests such as sodium assay (hyponatraemia), potassium assay (hyperkalaemia), urinary sodium test (increased sodium level), and plasma renin activity (increased plasma renin activity level) may be within the reach of laboratories in resource-limited countries. Owing to the severity of congenital adrenal hyperplasia, we think it should be a candidate for newborn screening programs in these countries.

### Inherited hyperbilirubinaemias

Congenital hyperbilirubinaemias arise from the absence of enzymes involved in bilirubin metabolism and transportation, especially uridine diphosphate glycosyltransferase. Some of these hyperbilirubinaemias include Crigler Najjar, Gilbert, Dubin-Johnson, and Rotors. These can be easily diagnosed by basic bilirubin determination (total and conjugated) and related to clinical presentation. A combination of blood and urine findings helps in the differential diagnosis.

### Newborn screening programmes

Newborn screening programmes are useful for the early detection of conditions with a presumptive period during which treatment can dramatically improve the outcome. These programmes are especially useful in resource-poor countries where facilities for managing ensuing complications are unavailable in delayed cases. While screening programmes are available in many developed countries, they are not available in many developing countries, and those that were previously available have degenerated. The assumption that the observed degeneration is due to a lack of advanced scientific equipment required for investigation is false as many IEMs can be detected using basic clinical chemistry laboratory facilities; this has been demonstrated in this brief article and supported by Brown and Lo^14^ and, more recently, Civallero, De Kremer and Giugliani.^11^ Even in the absence of tandem mass spectrophotometry in these countries, basic laboratory investigations can accomplish a great deal.

Inherited metabolic disorders such as congenital hypothyroidism (cretinism), phenylketonuria, and cystic fibrosis are commonly screened for in high-income countries like the United Kingdom. In developing countries, these disorders, as well as others such as maple syrup urine disease, galactosaemia, alpha-1-antitrypsin deficiency, UCDs, and L-N syndrome, can also routinely be investigated using basic laboratory facilities. For early diagnosis and to avoid the serious and irreversible consequences of IEM, close collaboration between clinics and metabolic laboratories, or just clinical laboratories, is critical.

A structured approach should be in place and may include targeted laboratory investigation and a comprehensive patient examination to assess drug use, feeding history, transfusion history, and family medical history. At the minimum, an IEM team consisting of clinicians (paediatricians), a clinical chemist or laboratory scientist, and a dietician must be available.

Interpretation of findings should take a multidisciplinary approach as this is one area where clinicians require a great deal of guidance from laboratory specialists. Many physicians who request laboratory tests are unfamiliar with the metabolic derangements associated with various IEMs. Brown and Lo^14^ propose that metabolic investigation reports should include age-related reference intervals for quantitative results. With a differential diagnosis derived from relevant abnormal and/or normal data, recommendations for further specialist investigations, and sufficient contact information for such institutions, there should be enough collaboration for clinicians to feel free to contact the laboratory if questions arise. Laboratory reports are best interpreted when clinical and relevant laboratory information are provided along with the test requisition, as is well-known in pathology and laboratory medicine. Results pointing to multiple IEMs should be correlated with the patient’s clinical and laboratory data to narrow the differential diagnosis before further investigation.^14,30^ It is recommended to conduct basic investigations that are useful in the diagnosis of IEMs as these are beneficial to the community (Table 3).^31^

**TABLE 3:** Basic investigations useful in the diagnosis of inborn errors of metabolism.

Test (blood)	Biochemical and clinical features
Glucose	Hypoglycaemia is a common presenting feature of many IEMs, notably organic aciduria and fatty acid oxidation disorders
Ammonia	Hyperammonaemia is a medical emergency requiring prompt treatment. It is common in organic acidurias and urea cycle disorders
Lactate	Increase in shock, hypoxia, and other metabolic abnormalities are important to exclude IEMs
Acid-base	Metabolic acidosis is common in many IEMs. Respiratory alkalosis may be an early indicator of urea cycle disorders and should prompt further investigations
Liver function tests	The liver function tests are commonly associated with liver abnormalities as in galactosaemia, Wilson’s disease, AAT deficiency, urea cycle defects with an increase in ALT and AST, and glycogenosis, which may cause an increase in ALP activity
Lipids	Increase in glycogen storage disorders
Uric acid	Von Gierke’s disease, Lesch-Nyhan’s syndrome, and galactosemia. Uric acid is decreased in molybdenum deficiency
CSF glucose	Markedly reduced glucose transporter 1
Iron and ferritin	An increase in hereditary haemochromatosis; may need other indices of iron metabolism
Ceruloplasmin	Low in both Wilson’s disease and Menkes disease
Copper (blood and urine)	Increased copper levels in urine in Wilson’s disease; low levels of copper in plasma in Menkes disease
Zinc	Low in acrodermatitis enteropathica due to deficiency of the intestinal zinc transporter
Alkaline phosphatase	Low in acrodermatitis enteropathica
Serum protein electrophoresis	Alpha (α)-band markedly reduced or completely missing in AAT deficiency

*Source*: Kruszka P, Regier D. Inborn errors of metabolism: From preconception to adulthood. Am Fam Physician. 2019;99(1):25–32.

AAT, alpha-1-antitrypsin; ALP, alkaline phosphatase; ALT, alanine transaminase; AST, aspartate transaminase; CSF, cerebrospinal fluid; IEMs, inborn errors of metabolism.

## Conclusion

Although the individual types of IEMs are rare, this category of diseases is common and often severe. Because severe morbidity and/or mortality can be avoided in some forms of IEM, it is critical to make accurate diagnoses early, especially in developing countries with limited complication management facilities. Though the definitive diagnosis of IEMs may require specialised laboratory investigations and interpretation, basic investigations that can be conducted in the average clinical chemistry laboratory in developing countries can provide valuable information for critical and early decision making in most cases. Referrals to specialised centres for further investigation can then be done. Appropriate interpretation of investigation findings requires interdisciplinary collaboration between clinical laboratory scientists and clinicians and is critical for the early diagnosis of patients with IEM and improved management of complex cases. A good team of paediatricians, clinical chemists (or laboratory scientists), and a dietician can be extremely useful. It is critical to emphasise that education and training for both health and non-health professionals are both essential and beneficial. Health facilities in developing countries need support from international organisations like the International Federation of Clinical Chemistry to improve current diagnostic capacity.
